# Redefining crop breeding strategy for effective use of nitrogen in cropping systems

**DOI:** 10.1038/s42003-022-03782-2

**Published:** 2022-08-16

**Authors:** Ignacio A. Ciampitti, Jean-Francois Briat, Francois Gastal, Gilles Lemaire

**Affiliations:** 1 Department of Agronomy, Kansas State University, Manhattan, KS USA; 2 Institute for Plant Sciences of Montpellier, Montpellier, France; 3 INRA, UE FERLUS, Les Verrines CS80006, 86600 Lusignan, France; 4 INRAE, 86600 Lusignan, France

**Keywords:** Plant breeding, Plant ecology, Plant physiology

## Abstract

In this Comment, Ciampitti et al. introduces a more relevant conceptual framework bridging soil and plant processes to untangle true gains of N for field crops rather than indirect progress merely based on yield.

## Teaser

More than a half-century after the initial conceptualization of nitrogen use efficiency, crop improvement progress for direct nitrogen gains remains an elusive target. A more relevant conceptual framework bridging soil and plant processes is needed for crop improvement and environmental stewardship.

Nitrogen (N) is a critical element to guarantee global food security and to reduce the environmental footprint of agriculture^1^. Maintaining a balance between N applied and crop N harvested is critical to minimize the consequences of global N losses^2^. The traditional metric, N use efficiency (NUE), refers to the responsiveness of crops to N fertilization^3,4^. However, a myriad of indices for estimating NUE have been proposed^5,6^, complicating the quantification of true N gains and comparison across cropping systems^7,8^. Recently, a perspective summary of NUE indices stressed the need to rethink this index relative to research goals while targeting global productivity and sustainability^9^.

## Traditional NUE: a metric for evaluating N fertilization efficiency

Crop NUE is traditionally defined as the ratio of the supplement of grain yield (Δ*Y*) or aboveground biomass (Δ*W*) to the supplement of N fertilizer application (ΔNf)^4^. NUE can be separated into N uptake efficiency (NupE) and N conversion efficiency (NCE):

1 $${{{{{\rm{NUE}}}}}}=\Delta {{Y}}/\Delta {{{{{\rm{Nf}}}}}}.$$

N uptake efficiency, NupE as the N taken up by the crop (ΔNup) per unit of ΔNf:

2 $${{{{{\rm{NupE}}}}}}=\Delta {{{{{\rm{Nup}}}}}}/\Delta {{{{{\rm{Nf}}}}}}.$$

N conversion efficiency, NCE as the Δ*W* per unit of ΔNup:

3 $${{{{{\rm{NCE}}}}}}=\Delta {{W}}/\Delta {{{{{\rm{Nup}}}}}}.$$

Harvest Index, HI is the increase in Δ*Y* to increase in Δ*W*:

4 $${{{{{\rm{HI}}}}}}=\Delta {{Y}}/\Delta {{W}}.$$

5 $${{{{{\rm{Therefore}}}}}},{{{{{\rm{NUE}}}}}}={{{{{\rm{NupE}}}}}}\times {{{{{\rm{NCE}}}}}}\times {{{{{\rm{HI}}}}}}.$$

The major drawback of these equations is that N uptake is co-regulated by both soil N availability and potential plant growth rate^10^, which determine root N absorption capacity. Thus, the dissection of NUE into NupE and NCE does not allow a relevant analysis of mutual roles of soil and/or plant processes. Consequently, NUE cannot be interpreted as a general property of a given genotype to efficiently use all its N resources.

## Toward a re-definition of NUE for crop breeding objectives

**Fig. 1:**
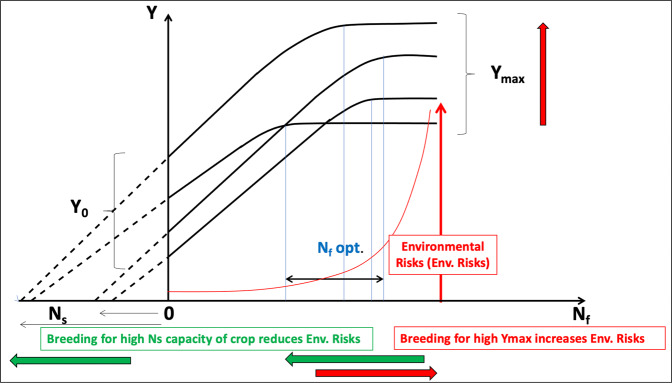
Schematic representation of crop yield (*Y*) response to N fertilizer application rate (Nf). The response curve *Y* = f(Nf) is asymptotic and dY/dNf declines as Nf increases. Thus, *Y* increases from a value of *Y*_0_ (yield without N fertilization) until a maximum yield (*Y*_max_) is reached for an optimum value of Nf = Nf_opt_. The environmental risk for N losses is directly linked to Nf application and increases rapidly as Nf approaches and exceeds Nf_opt_. Linear extrapolation of dY/dNf allows the estimation of an “apparent” contribution by soil N supply (Ns).

It is imperative to re-define NUE for crop breeding objectives, maximizing grain yield (*Y*_max_), mainly driven by application of N fertilizers (Nf) and the contribution by soil N supply (Ns), while minimizing environmental N losses^11^ (Fig. 1).

Therefore, if the goal is to assess the ability of a crop to produce yield or biomass per unit of N, then Eq. (1) should be reformulated as:

6 $${{{{{\rm{NUE}}}}}}=\Delta {{Y}}({{{{{\rm{orW}}}}}})/\Delta ({{{{{\rm{Nf}}}}}}+{{{{{\rm{Ns}}}}}}).$$

However, Ns is also affected by Nf (as demonstrated by ^15^N studies^12^) and controlled by the plant itself. Therefore, attempts to compute Ns + Nf as an external resource are not relevant, as Ns and Nf are intertwined.

Figure 1 illustrates two strategies for increasing crop performance in N use:

1. Breeding for high *Y*_max_ (or *W*_max_) at high N supply, leading to an increased slope dY/dNf, but with increased environmental risks linked to higher Nf applications.
2. Breeding for high crop N uptake capacity at low N supply, reducing the crop dependency on Nf and minimizing environmental risks

The first strategy is clearly to maximize NCE, while the second strategy is oriented toward maximizing NupE. However, the asymptotic response of Y (or W) to Nf would create a trade-off between NCE and NupE. This feature illustrates the impossibility to separately analyze NupE and NCE as relevant crop traits for NUE.

## Holistic N approach considering the soil-plant system

**Fig. 2:**
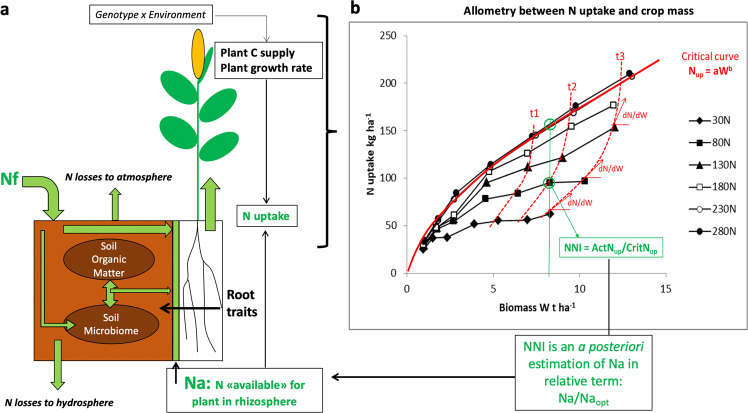
Schematic representation of soil-plant system. **a** Na, the N available for plant remains a virtual entity due to the fractal geometry of the rhizosphere. **b** Allometry between plant N uptake (Nup) and crop mass (W), Nup = aW^b^, reflecting (i) the feedback control of the rate of root N absorption by both N availability (Na) and potential plant growth rate in time (dW/dt) and (ii) the diminishing of the crop N demand as plant mass increase dNup/dW = ab W^b-1^ leading to crop N dilution process. This allometry implies that both N uptake efficiency (NupE) and N conversion efficiency (NCE) increases as crop W increases. NNI nitrogen nutrition index, ActN_up_ (Actual N uptake)/CritN_up_(Critical N uptake), Nf N fertilizer.

The soil-plant system is an integrated, auto-regulated system within which plants interact with soil to determine the N available for root absorption^13^ (Na). Thus, as indicated in Fig. 2a, Na is the relevant bridge variable connecting soil to plant sub-systems but depending upon: (1) soil characteristics (organic matter and microbiome interactions), (2) plant root traits (density and architecture^14^) allowing forage of NH_4_^+^ and NO_3_^−^ through active root development^15^, and (3) C substrate deposition and exudation within the N mineralization-organization turnover^16,17^.

As illustrated in Fig. 2b, N uptake rate is regulated by Na within the rhizosphere but is also feedback-regulated by plant growth capacity^10^. This co-regulation of N uptake leads to an allometry of this process with W^18^, with the critical Nup = aW^b^ curve defined as the minimum N uptake required for maximum W. This critical curve has been demonstrated as stable across genotype × environment (G × E) scenarios in maize (*Zea mays* L.) and tall fescue (*Festuca arundinacea* Schreb.) crops^18,19^, but divergent for C3 versus C4 species^20^. When N becomes limiting, the intensity of crop N deficiency can be quantified by the distance of any data point (Nup-W) to the critical curve, i.e., the Nitrogen Nutrition Index (NNI)^21^. Determination of NNI, as proposed in recent literature^22^, is a prerequisite for deciphering NUE and its efficiency terms.

## Metrics for effective use of N in field crops

The traditional NUE (Eq. (1)) index is a proper metric of the crop responsiveness to Nf. However, if the objective is to evaluate agro-ecological crop performance and provide relevant plant traits for breeding programs, the determination of crop NNI will be necessary to clearly separate plant traits related to:

1. increasing plant N demand as a consequence of plant crop W capacity.
2. increasing plant capacity to satisfy its own N demand via its effect on Na.

The determination of NNI facilitates this distinction and disentangles the confusing estimation of both NCE and NupE terms in NUE data^22–24^.

## New breeding strategies for improving acquisition of resources

Most NUE-focused plant breeding programs for different crop species utilize non-limiting, or at least large, Nf applications. As established for maize^11^, breeding progress achieved through selectively increasing *W*_max_ or *Y*_max_ can have a small effect on *Y*_0_ (Fig. 1) but necessitate higher N application rates to achieve *Y*_max_, potentially increasing the risk for N losses. Concurrently, crop sensitivity to N deficiency is enhanced when Ns is low.

This concept evolution occurs simultaneously with the development of new strategies to improve resource acquisition capacity of plant roots. A number of genes have been identified both in model and crop plants which facilitate enhanced nutrient acquisition^25^. Meta-analysis and allele combination analysis indicated that root system depth and root spreading angle are valuable candidate traits for improving grain yield by pyramiding favorable alleles^26^. Genome-wide association studies and quantitative trait loci are powerful tools for understanding genetic variation of root architecture and delivering markers to assist new breeding strategies to facilitate genetic improvement of water/nutrient efficiencies.

## A forward-facing outlook

In summary, the root availability of N in the rhizosphere is clearly the bridge variable to connect soil and plant processes. In addition, NNI is an a posteriori proxy of the ability of plant roots to capture and utilize available rhizospheric N. Thus, NNI determination is fundamental to untangle true gains of NUE rather than trivial pseudo efficiency improvements solely based on yield. The integration of NNI in crop breeding programs can be more rapidly ingested (high-throughput phenotyping^23,27^) with the utilization of new technologies (e.g., sensors, satellite) under varying G × E × M conditions^28,29^.

Although the concept presented here is mainly focused on N, the foundation and framework could be extended to other nutrients to improve our understanding in a more holistic approach of nutrient use efficiency at a broader soil-plant system scale. Lastly, crop improvement for effective use of nutrients will require the integration of key scientists (i.e., agronomists, crop physiologists, breeders)^30^ to realize the potential benefits of direct selection and for rapid progress^31^ to overcome this complex challenge and address food security goals in a more sustainable way.

## Data Availability

The code used for this analysis is available from the corresponding author on request.
